# Overview of Four Functional Classification Systems Commonly Used in Cerebral Palsy

**DOI:** 10.3390/children4040030

**Published:** 2017-04-24

**Authors:** Andrea Paulson, Jilda Vargus-Adams

**Affiliations:** Cincinnati Children’s Hospital Medical Center, Cincinnati, OH 45229, USA; jilda.vargus-adams@cchmc.org

**Keywords:** cerebral palsy, classification, severity, function, GMFCS, MACS, CFCS, EDACS

## Abstract

Cerebral palsy (CP) is the most common physical disability in childhood. CP comprises a heterogeneous group of disorders that can result in spasticity, dystonia, muscle contractures, weakness and coordination difficulty that ultimately affects the ability to control movements. Traditionally, CP has been classified using a combination of the motor type and the topographical distribution, as well as subjective severity level. Imprecise terms such as these tell very little about what a person is able to do functionally and can impair clear communication between providers. More recently, classification systems have been created employing a simple ordinal grading system of functional performance. These systems allow a more precise discussion between providers, as well as better subject stratification for research. The goal of this review is to describe four common functional classification systems for cerebral palsy: the Gross Motor Function Classification System (GMFCS), the Manual Ability Classification System (MACS), the Communication Function Classification System (CFCS), and the Eating and Drinking Ability Classification System (EDACS). These measures are all standardized, reliable, and complementary to one another.

## 1. Introduction

Cerebral palsy (CP) is the most common physical disability in childhood [1]. CP comprises a heterogeneous group of disorders that are the result of a non-progressive disruption or injury that occurred during fetal brain development or within the first two years of life [2]. This disruption can result in spasticity, dystonia, muscle contractures, weakness and difficulty in coordination that ultimately affects the ability to control movements [3]. Resultant activity limitations may affect gross motor movements, fine motor movements, speech and communication, as well as eating and drinking. Traditionally, CP has been classified using a combination of the motor type and the topographical distribution. Motor types include terms like spastic, hypotonic, ataxic, dyskinetic or mixed. The topographic classifications include the limbs that are affected, namely diplegia (or diparesis), tri-, tetra-, quadri- or hemiplegia. CP severity is frequently described subjectively in terms of mild, moderate or severe [4,5]. Imprecise terms such as these tell very little about what a person is able to do functionally. Moreover, when a large range of ability and performance can be encapsulated by the same term (such as moderate spastic quadriplegia CP), providers struggle to accurately interpret communications from one another. In research, this can create subject groups that are very heterogeneous.

Over the last 20 years, clinicians and researchers have created classification systems using a simple ordinal grading system of functional capacity, allowing a more precise discussion between providers. These classification systems use a common language to describe function. They do not describe potential improvements or assess underlying etiology. This paper provides an overview of four common functional classification systems used in cerebral palsy: the Gross Motor Function Classification System (GMFCS), the Manual Ability Classification System (MACS), the Communication Function Classification System (CFCS), and the Eating and Drinking Ability Classification System (EDACS). These measures are standardized, reliable, and complementary to one another.

## 2. Classification Measures

### 2.1. Gross Motor Function Classification System (GMFCS)

**Figure 1:**
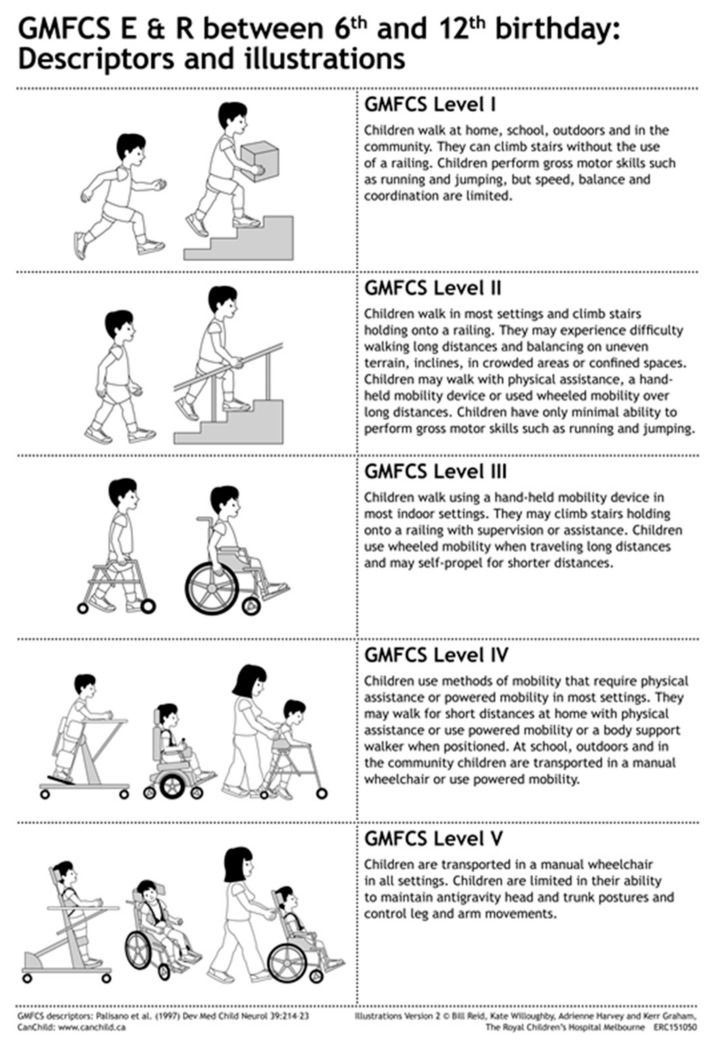
Five Gross Motor Function Classification System (GMFCS) Expanded and Revised levels, as depicted for children ages 6–12 years. Reproduced with permission [8].

The GMFCS is the most established and recognized of the functional classification measures in CP. The GMFCS is a simple, five-level, ordinal grading system created to describe the gross motor function of an individual with CP. First described in 1997 by Palisano et al. [6], the GMFCS provides a common language for a practitioner that is meaningful, quick and easy to use. The GMFCS describes self-initiated movement and use of assistive devices (walkers, crutches, canes, wheelchairs) for mobility during an individual’s usual activity. Following research to stratify typical motor function for children with CP, the authors concluded that a five-level classification system worked well to discriminate clinically meaningful distinctions in motor function. This classification system was initially designed to be used with children 2–12 years of age [6,7]. The GMFCS was later expanded and revised in 2007 to include ages 12–18, as well as to increase descriptors and differentiations for the levels based on the child’s age, while taking into account developmental milestones [8]. These more detailed and age appropriate descriptors allow a more accurate classification. A pictorial representation can be found in Figure 1, for ages six through 12.

Based on the GMFCS revised and expanded version, an individual classified in GMFCS I is able to walk without limitations. Individuals less than two years of age are able to crawl on hands and knees, pull to stand, cruise when holding onto furniture, and are able to attain independent walking between the age of 18 months and two years. Between two and four years of age, skills include sitting independently and transitioning between sitting and standing independently. Between age four and six years, the individual in GMFCS I is able to walk indoors and outdoors independently, climb stairs, and start to run and jump. Between the ages of six and twelve, additional abilities include walking up and down curbs, walk community distances, negotiate stairs without railings, and run and jump (which may include some limitations). Between age 12 to 18, the abilities are the same as age 6 to 12.

A child classified in GMFCS II can walk with limitations. Limitations may include balance or endurance, use of a hand-held mobility device prior to age 4, use of a railing on stairs, or an inability to run or jump. GMFCS II functioning may result in the use of wheeled mobility for long distances. A child before the age of two can sit with upper extremity support, crawl on their stomach, and may be able to pull-to-stand or cruise with support. Between ages 2 and 4, the child can transition into and out of sitting without support, can sit without support (but may need to use their upper extremities for balance), can crawl on hands and knees, cruise with support and walk with a mobility device. Between the ages of four and six, the individual can transition into and out of standing without support, walk short flat distances without an assistive device, do stairs with the railing, but is unable to run or jump. From age 6 to 12, the individual can walk in most terrains but has limitations with distance or uneven surfaces, may use wheeled mobility for long distances, can do stairs with the railing, but is able to do minimal or no running and jumping. Between ages 12 and 18, the abilities are the same as age 6 to 12, but a handheld mobility device may be used for safety.

A child classified in GMFCS III can often walk with a hand-held mobility device indoors, but use wheeled mobility in the community and for longer distances. GMFCS III level functioning indicates the ability to sit with little to no external support and to stand to complete transfers. Children in GMFCS III who are less than 2 years old can roll and occasionally crawl forward when lying on their stomach, as well as sit with some low back support. Between ages 2 and 4, a child can “W” sit on the floor with some help getting into the position, crawl on his/her stomach or creep on all fours, and may pull-to-stand and walk short distances using a handheld mobility device (walker or gait trainer) with some assistance for maneuvering. From age 4 to 6, a child can sit in a standard chair but may require extra support to allow full upper extremity function, walk with a handheld mobility device and do stairs with assistance; typically, wheeled mobility is used for longer distances. In GMFCS III, children aged 6 to 12 walk with a handheld mobility device indoors and use wheeled mobility (either manual or powered) for distance, require assistance to move between floors, sitting and standing, and negotiate stairs with assistance. For ages 12 to 18, the abilities are the same as age 6 to 12, but more variability is demonstrated in primary mobility preferences.

An individual classified in GMFCS IV can sit supported, but self-mobility is limited, often being transported in a manual wheelchair or using powered mobility. Children before the age of 2 have head control and can roll, but require truncal support to sit. Between ages 2 and 4, a child in GMFCS IV can sit with upper extremity support, require assistance to transition into sitting, and may require adaptive equipment for sitting or standing. At this age, some self-mobility is possible through rolling or stomach crawling short distances, but reciprocal leg movement is not present. From age 4 to 6, children require adaptive equipment for trunk control to allow sitting and assistance to move between positions. Children may walk short distances with a mobility device and with assistance, and use wheeled mobility for distances, and/or be independent with powered mobility. Children in GMFCS IV and ages 6 to 12 require adapted seating and assistance with transfers, and utilize wheeled power mobility independently or manual mobility with assistance in most settings. Many children can have independent floor mobility with crawling or rolling, or may walk short distances with assistance. For ages 12 to 18, the abilities are the same as ages 6 to 12.

Children classified in GMFCS V have more severe limitations with head and trunk control and self-mobility is only possible using a power wheelchair. Children in GMFCS V before the age of 2 do not have independent head or trunk control and require assistance to roll. Between ages 2 and 4, a child has no independent movement and requires assistance for transport using manual mobility devices. Adaptive equipment is required for sitting and standing, but function is still limited. It is possible to become independent using power mobility with additional adaptations. From age 4 onwards, the abilities of children in GMFCS V are stable with a need for complete assistance with transfers emerging after age 6 [8].

#### 2.1.1. Inter-Rater and Test-Retest Reliability

During the creation of the GMFCS, Palisano et al. found the inter-rater reliability to have a moderate agreement with a kappa (*k*) of 0.55 in children <2 years of age and excellent agreement with a kappa of 0.75 in children 2–12 years of age [6]. This strong inter-rater reliability supports the use of the GMFCS as a classification of gross motor function in children ages 2–12. Therefore, the authors concluded that classification under the age of 2 years is possible, but should be done with caution. The expanded and revised GMFCS underwent validation testing using group consensus methods and was found to be valid with >80% agreement of participants [9]. Additional studies assessing the GMFCS inter-rater reliability have been completed. Bodkin et al. [10] compared two experienced pediatric physical therapists when assessing a patient via videotape, and found the inter-rater reliability was 0.84. The authors also reported that the GMFCS level assigned remained stable over the two years of the study [10].

If the GMFCS is to be a useful classification system, it needs to be relatively stable over time, as a child with CP changes with age and development. A retrospective review was completed in 2000, in order to assess the reliability and stability of the GMFCS over time, and found an inter-rater reliability of 0.93 and a test-retest reliability of 0.79 [11]. The authors also report that the positive predictive value of the GMFCS at 1–2 years of age, to predict walking by 12 years of age, was 0.74 and the negative predictive value was 0.90 [11]. Palisano et al. also reported excellent stability of the GMFCS over time with a weighted kappa coefficient of 0.84 for children younger than 6 years of age, and 0.89 for children older than 6 years [12]. Additionally, McCormick et al. reported the stability of the GMFCS from age 12 into adulthood, with a weighted kappa of 0.895. The inter-rater reliability was 0.978. The positive predictive value of the GMFCS at age 12 for a current wheelchair user to remain a wheelchair user as an adult was high, with a weighted kappa of 0.96, and for a person walking without mobility aid to remain without a mobility aid, kappa was 0.88 [13]. Morris et al. found that family-reported GMFCS level was also a reliable method for measuring gross motor function in children between the ages of 6–12 years [14]. Therefore, not only can medical providers accurately classify children rapidly and accurately in clinical settings using the GMFCS, but parents and caregivers are also effective at selecting the GMFCS for children.

### 2.2. Manual Ability Classification System (MACS)

**Figure 2:**
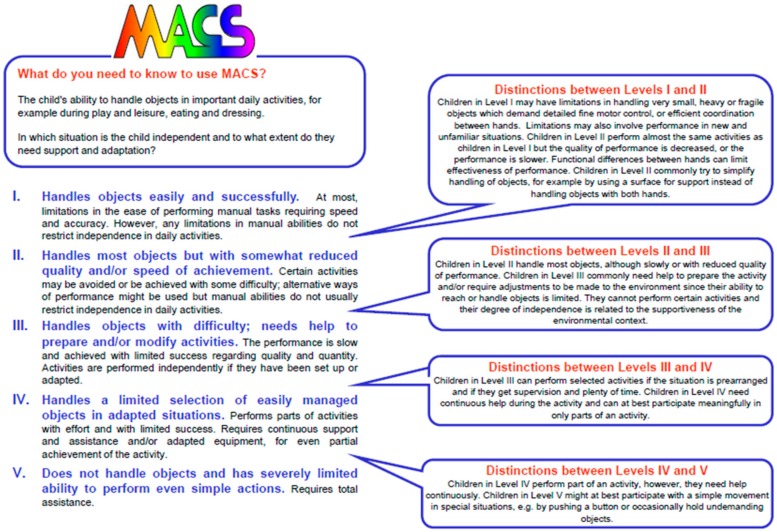
Summary of the five Manual Ability Classification System (MACS) levels. Reproduced with permission [15].

The Manual Ability Classification System (MACS) was developed by Eliasson et al. in 2006, specifically to be an upper extremity analogue of the GMFCS. The MACS is also a simple, five-point ordinal classification system, analogous and complementary to the GMFCS, and was designed for use in children ages 4–18 years. The MACS is a validated measure in cerebral palsy that can be used to classify a child’s typical use of both hands and upper limbs; it is not meant to reflect best use or individual hand function [15]. Figure 2 shows a portion of the MACS scale.

An individual classified as MACS I is able to handle objects easily with the possibility of some limitations with very small, fragile or heavy objects, or limitations with fine motor control, but these limitations do not restrict independence in daily activities. MACS II classification indicates a decreased level of performance when handling objects; the performance may be slower and may be impacted by asymmetric hand function. An individual may use alternative ways to handle objects, but remains independent in daily activities. An individual in MACS III handles objects slowly and often with limited success, requiring assistance or set-up for activities. Some activities can be completed independently with appropriate adaptations and set up, but other activities cannot be adequately performed without assistance. MACS IV functioning indicates a need for continuous support and assistance or the use of adapted equipment to complete only a part of a daily activity, with an inability to complete the full activity. Individuals in MACS V do not handle daily objects and may be able to participate minimally with simple movements or may require total assistance.

#### 2.2.1. Intra-Class Correlation

During development of the MACS, it underwent reliability testing between professionals, as well as between a professional and a parent. The MACS has an intra-class correlation coefficient (ICC) between professionals of 0.97 and between a professional and parent of 0.96, indicating outstanding reliability [15]. Morris et al. explored parent MACS ratings and also reported the MACS to have high reliability, with ICC of 0.7–0.9, indicating agreement in classification between families and professionals. The authors therefore concluded that the MACS is a reliable and valid classification method for manual ability in children with CP [16].

In 2013, Ohrvall et al. completed a study that assessed the stability of the MACS over time and found that the ICC was 0.97 and two ratings over a one-year interval had an 82% agreement. The stability for two ratings performed at a 3–5-year interval had an ICC of 0.96 and an agreement of 78%. When MACS was followed over four ratings, 70% of children remained at the same rating, and the score was consistent for younger and older children, indicating that the rating stability is not influenced by age [17].

#### 2.2.2. Inter-Observer Reliability

In 2009, Plasschaert et al. published a study that assessed the inter-observer reliability of the MACS when used in children ages 1–5 with cerebral palsy. They found the inter-observer reliability of the MACS to have a linear weighted kappa (*k*) of 0.62. When assessing only children <2 years of age, the *k* = 0.55, which is a moderate reliability, but when assessing children 2–5 years of age the *k* = 0.67, demonstrating good reliability. The authors concluded that classification between 2–5 years of age is possible, but that classification at age <2 should be done with caution, and recommended an additional tool be created to better classify these young children [18].

### 2.3. Mini-MACS

The Mini-MACS was developed by Eliasson et al. in 2016 and is designed to classify children`s manual ability for those aged 4 years or younger. The Mini-MACS underwent reliability testing between professionals, as well as between a professional and a parent. The Mini-MACS has an ICC between professionals of 0.97, and between a professional and parent of 0.90, indicating good reliability and validity. The wording of the Mini-MACS is very close to that of the MACS, with descriptions more relevant for younger ages when compared to the MACS [19].

In Mini-MACS I, children can have slight limitations in handling objects with higher levels of coordination, and may need some assistance compared to their peers. At Mini-MACS II, children have more difficulty with everyday objects, require practice, often use an alternative approach, and require additional assistance compared to peers. Children in Mini-MACS III can handle easy objects independently for short periods of time but requires additional time and often assistance to support the object. In comparison, Children in Mini-MACS IV can perform simple actions when handling an object, for example grasping and releasing an item, but require constant support for handling. Children in Mini-MACS V may be able to complete simple movements with constant support, for example pushing a switch or button [19].

### 2.4. Communication Function Classification System (CFCS)

Thirty one to 88% of individuals with CP are estimated to have a concomitant communication disorder [20,21]. The CFCS was developed by Hidecker et al. in 2011 and is a valid measure in cerebral palsy that assesses everyday communication (not optimal communication) [22]. The CFCS is a simple, five-point ordinal classification system, and was designed to be analogous and complementary to the GMFCS and MACS. Being an effective communicator requires both sending and receiving information, and the CFCS accordingly assesses both how information is expressed and how it is received. The CFCS allows all methods of communication (e.g., vocalizations, manual signs, eye gaze, pictures, communication boards, speech generating devices) to be included when assessing an individual’s classification. In this way, the CFCS is inclusive and descriptive for individuals who do not vocalize to communicate. The CFCS also takes into account familiar and unfamiliar communication partners [22].

An individual who communicates at a CFCS level I is known as an “effective sender and receiver with unfamiliar and familiar partners”. The individual is able to communicate at a comfortable pace, send and receive information with familiar and unfamiliar partners, and any misunderstandings are easily corrected. An individual who communicates at a CFCS level II remains an effective communicator, but the pace of communication is slower. An individual at this level is known as an “effective but slower paced sender and/or receiver with unfamiliar and/or familiar partners”. The primary difference between a level I and level II communication functioning is the pace of the communication; however, both are effective at sending and receiving information.

An individual who communicates at a CFCS level III is known as an “effective sender and receiver with familiar partners”. The primary difference from CFCS I and II is that communication is effective with familiar partners, but is usually not effective with an unfamiliar partner, due to decreased intelligibility. At CFCS level IV, an individual is an “inconsistent sender and/or receiver with familiar partners”. An individual may occasionally communicate with familiar partners, but it is the inconsistency of this interaction that makes the distinction for level IV compared to level III. An individual who communicates at a CFCS level V is a “seldom effective sender or receiver even with familiar partners”. This is in contrast to a level IV where there is inconsistency with communication; a level V consistently has ineffective communication.

#### 2.4.1. Inter-Rater and Test-Retest Reliability

During development of the CFCS, it underwent evaluation for test-retest reliability, as well as inter-rater reliability. The test-retest reliability was reassuring at 0.82. The inter-rater reliability was found to be 0.66 when two professionals were classifying an individual and 0.49 when a professional and a parent were assigning a classification. Professional inter-rater reliability improved to 0.77 for individuals over the age of three years. Overall, the CFCS had good test-retest reliability and professional inter-rater reliability, with slightly less parent-professional inter-rater reliability [22]. The CFCS has been translated into Dutch and Farsi and these measures have also demonstrated good reliability [23,24].

### 2.5. Eating and Drinking Ability Classification System (EDACS)

In addition to impairments of gross motor function, fine motor function and communication, individuals with CP can also have impairments in eating and drinking, as a result of difficulty with motor control. Between 27% and 90% of people with CP are estimated to have some degree of difficulty with eating or drinking [25].

The EDACS was developed by Sellers et al. in 2014, and is a valid measure to assess eating and drinking ability for children with CP, ages 3 and older [26]. This classification is a simple five-point ordinal system, designed to be analogous and complementary to the GMFCS, MACS and CFCS. The EDACS assesses eating and drinking safety (aspiration and choking) as well as efficiency (amount of food lost and time taken to eat). The EDACS also adds an additional three-point ordinal scale that addresses the amount of assistance a person needs: independent; requires assistance; or dependent for eating and drinking [26].

An individual in EDACS I can independently eat and drink safely and efficiently, no different from their peers. A cough or gag could be present for very challenging textures. An individual in EDACS II eats and drinks safely, may have some limitations in terms of food loss, and generally requires more time to complete a meal than peers. A cough may be present with fast flowing liquid or large quantities of food. Both EDACS I and II include the ability to eat a wide range of textures, consistent with their peers. At EDACS III, an individual eats and drinks with some limitation to safety as well as efficiency. Hard lumps of food may be difficult to manage, or choking may be a risk. Many individuals in EDACS III primarily eat pureed or mashed foods with occasional soft chewing of limited textures. A cough may be present with fast flowing liquid or a large bolus of food.

In comparison, an individual in EDACS IV or V cannot swallow food and drink without risk of aspiration. At EDACS IV, there are significant limitations with safety; however, the risks of aspiration can be managed and oral feeding is possible. An individual in EDACS IV will eat smooth purees or well mashed foods only. The coordination of swallowing and breathing may be difficult and there may be signs of aspiration. An individual in EDACS V is unable to eat or drink safely, and relies on tube feeding for nutrition; he or she may manage small tastes or flavors. Those in EDACS V are at high risk for aspiration.

The EDACS also assesses the level of assistance required for feeding, via a simple three-point classification system (independent; requires assistance; or totally dependent). To obtain the “independent” classification, an individual must bring food and drink to their mouth without assistance. If an individual uses adaptive equipment or requires another individual to use an adaptive equipment when assisting them in bringing food or drink to their mouth, they would be classified as “requiring assistance”. A classification of “totally dependent” indicates that the individual requires another person to bring the food or drink to their mouth [26].

#### 2.5.1. Intra-Class Correlation

As the EDACS was developed, it also underwent agreement testing and reliability testing between speech and language pathologists (SLP), as well as between an SLP and a parent. The EDACS scored 78% on absolute agreement between two SLPs, with an ICC coefficient of 0.93. When a discrepancy occurred, it was by a single EDACS level with only one exception that was of more than a single level. The EDACS had an absolute agreement of 58% when classified between an SLP and a parent, with an ICC of 0.86. Parents either agreed with the SLP or scored their child one classification level more able than the SLP. Overall, the EDACS had good agreement testing and reliability testing with slightly less parent-SLP agreement, making this a valid and reliable classification system [26].

## 3. Discussion

With the development of the four instruments discussed in this review, providers have a framework for a common language to better describe and communicate about the vastly heterogeneous functional abilities of individuals with CP. This common language is important for quick and accurate transmission of information from one care provider to the next or from care provider to caregiver. Using the GMFCS, MACS, CFCS, and EDACS, descriptions of patients are more accurate and meaningful than solely traditional topographic descriptions, but can be used in conjunction with these descriptors as a complementary inclusion. In addition to facilitating communication between providers, these classification systems also standardize populations for clinical research. A summary of the five classification levels can be found in Table 1.

Additionally, providers should remember that ongoing development throughout childhood requires them to note the age of each individual when assigning a functional level. The use of these various classification systems should be adopted as a standard care for the diagnosis and management of all children with CP.

Not only do the GMFCS, MACS, CFCS, and EDACS refine and simplify descriptions of patients with CP, they have done this in a robust fashion. These classifications have been found to be reliable and stable over time. With modest training, these measures can be assessed rapidly and accurately—and not just by professionals, but also often by family members. The simplicity, yet richness, of these classifications have transformed the description and quality of care for children with cerebral palsy.

## Figures and Tables

**Table 1:** Five classification levels of the Gross Motor Functional Classification System (GMFCS), the Manual Ability Classification System (MACS), the Communication, Function Classification System (CFCS) and the Eating and Drinking Ability Classification System (EDACS).

	GMFCS	MACS	CFCS	EDACS
**I**	Walks without limitation	Handles objects easily and successfully	Effective sender and receiver	Eats and drinks safely and efficiently
**II**	Walks with limitations (no mobility aid by 4yo)	Handles most objects with reduced speed/quality	Effective but slow paced sender and receiver	Eats and drinks safely but with some limitations to efficiency
**III**	Walks with hand-held mobility device	Handles objects with difficulty, help to prepare or modify activity	Effective sender and receiver with familiar partners	Eats and drinks with some limitations to safely; there may also be limitations to efficiency
**IV**	Self-mobility with limitations, may use power	Handles limited number of objects in adapted setting	Inconsistent sender and receiver with familiar partners	Eats and drinks with significant limitations to safety
**V**	Transported in manual wheelchair	Does not handle objects	Seldom effective sender and receiver with familiar partners	Unable to eat or drink safely, consider feeding tube
